# J-shaped association between serum albumin levels and long-term mortality of cardiovascular disease: Experience in National Health and Nutrition Examination Survey (2011–2014)

**DOI:** 10.3389/fcvm.2022.1073120

**Published:** 2022-11-29

**Authors:** Xu Li, Yue Zhang, Yi He, Ke-Xin Li, Ruo-Nan Xu, Heng Wang, Ting-Bo Jiang, Wei-Xiang Chen, Yong-Ming He

**Affiliations:** Division of Cardiology, The First Affiliated Hospital of Soochow University, Suzhou, Jiangsu, China

**Keywords:** albumin levels, cardiovascular disease, mortality, J-shaped association, NHANES

## Abstract

**Background:**

Cardiovascular disease (CVD) is a constellation of heart, brain, and peripheral vascular diseases with common soil hypothesis of etiology, and its subtypes have been well-established in terms of the albumin-mortality association. However, the association between albumin and the mortality of CVD as a whole remains poorly understood, especially the non-linear association. We aimed to investigate the association of albumin levels with long-term mortality of CVD as a whole.

**Materials and methods:**

This study included all CVD patients who participated in the National Health and Nutrition Examination Survey (NHANES 2011–2014). CVD was defined as coronary heart disease, stroke, heart failure, or any combination of these two or three diseases. Serum albumin was tertile partitioned: tertile 1, <4.1; tertile 2, 4.1–4.3; and tertile 3, >4.3 g/dl. COX proportional hazards model was used to assess the association between the serum albumin levels and CVD mortality. Restricted cubic spline (RCS) curves were used to explore the non-linear relationship.

**Results:**

A total of 1,070 patients with CVD were included in the analysis, of which 156 deaths occurred during a median 34 months of follow-up. On a continuous scale, per 1 g/dl albumin decrease was associated with an adjusted HR (95% CI) of 3.85 (2.38–6.25). On a categorical scale, as compared with tertile 3, the multivariable adjusted hazard ratio (95% CI) was 1.42 (0.74–2.71) for the tertile 2, and 2.24 (1.20–4.16) for the tertile 1, respectively, with respect to mortality. RCS curve analysis revealed a J-shaped association between albumin and CVD mortality.

**Conclusion:**

A J-shaped association between low serum albumin levels and increased long-term mortality of CVD has been revealed. This J-shaped association’s implications for CVD prevention and treatment are deserving of being further studied.

## Introduction

Cardiovascular disease (CVD) mainly includes coronary heart disease (CHD), stroke, heart failure (HF), and also covers other cardiovascular conditions (1). Accumulating evidence has shown that low albumin level is a well-established risk factor for its subtypes (2), such as stable CHD (3), acute coronary syndrome (4–9), acute and chronic HF (10–18), and ischemic stroke (19–21). In patients with acute coronary syndrome, as compared with albumin ≥3.5 g/dl, those with albumin <3.5 g/dl were associated with a 2.8-fold greater risk of adverse outcomes (death, acute heart failure, cardiogenic shock, and reinfarction) (4); A 3-year follow-up study of patients with first-onset acute myocardial infarction found per 1 g/dl decrease was associated with an unadjusted hazard ratio (95% CI) of 4.11 (3.17–5.33) (6); Hypoalbuminemia (≤3.4 g/dl) predicted 1-year mortality in acute decompensated heart failure [HR (95% CI), 2.05 (1.10–3.81)], and also predicted one and a half year- mortality in patients with chronic heart failure [HR (95% CI), 5.74 (4.08–8.07)], when compared with non-hypoalbuminemia (11, 14). In addition, a prospective community-based cohort study has demonstrated that low albumin level is an independent predictor of cerebro-cardiovascular death [HR (95% CI), 5.26 (1.59–16.67)] (19). However, cardiovascular disease is a constellation of heart, brain, and peripheral vascular diseases with common soil hypothesis of etiology, and the association between albumin and mortality in CVD as a whole remains poorly understood, especially the non-linear association. We aimed to investigate the association between albumin levels and long-term mortality of CVD as a whole.

## Materials and methods

### Study population

We obtained publicly available data from the National Health and Nutrition Examination Survey (NHANES). NHANES, with a probability sampling design to represent the civilian non-institutionalized population of the United States, was designed to assess the health and nutritional status of the general population. The NHANES survey included not only demographic, socioeconomic, dietary, and health-related interview data, but also examination data for medical, dental, and physiological measurements. The survey protocol was approved by the National Institute of Health Research Ethics Review Board, and all participants signed and provided informed consent. For this study, we included patients who were diagnosed with CVD in the continuous two cycles of NHANES survey of 2011–2014. The study population was divided into three groups according to the tertiles of albumin levels (<4.1, 4.1–4.3, and >4.3 g/dl).

### Outcomes, exposures, covariates, and their definitions or measurements

The primary outcome was CVD mortality. CVD diagnosis includes CHD, stroke, heart failure, or any combination of these two or three diseases. The exposure was the serum albumin levels. We adjusted for covariates with respect to demographics, lifestyle, chronic diseases, and blood indicators, including age, sex, ethnicity, BMI, pulse, drinking, smoking, hypertension, diabetes mellitus, albumin, triglycerides, LDL-C, and HDL-C. The definition of smoking referred to New Zealand standards (22). Hypertension was measured as SBP ≥ 140 and or DBP ≥ 90 mmHg or as self-reported one by asking the question, “Has a doctor or other health professional ever told you that you have hypertension?”. The diagnosis of diabetes referred to the most recent ADA criteria (A1C ≥ 6.5% or FPG ≥ 7.0 mmol/L or 2-h OGTT ≥ 11.1 mmol/L or a random plasma glucose ≥ 11.1 mmol/L) (23). BMI was evaluated by body mass (kilograms) and body height (m^2^). Albumin levels were measured by DcX800 method. Triglycerides and HDL-C were measured by the Roche/Hitachi Modular P Chemistry Analyzer (Mod P) in Mobile Examination Centers (MECs). LDL-C was calculated from measured values of total cholesterol, triglycerides, and HDL-C according to the Friedewald calculation. Information on all variables and their measurement methods are publicly available on the NHANES website (24).

### Statistical analysis

Frequency distribution diagram was used to investigate the distribution of albumin. Independent groups were compared using the Wilcoxon rank test or likelihood ratio test as appropriate. Kaplan-Meier curves were used to compare survival differences between different albumin tertiles. COX proportional hazards model was used to assess the association between albumin levels and CVD mortality. Restricted cubic spline (RCS) curves were used to explore the non-linear relationship between albumin levels and CVD mortality. Subgroup analyses were performed to test whether the association between albumin levels and CVD mortality was consistent in different groups. Interactions between albumin and other covariates were examined. Adjusted covariates included age, sex, ethnicity, BMI, pulse, drinking, smoking, hypertension, diabetes mellitus, triglycerides, LDL-C, and HDL-C. Multiple imputation (Chained equations, 25 times) was used to fill in the missing values. All analyses were conducted using Stata 15.1 (Stata Corp, TX, USA). All tests were two-sided. Statistical significance was considered when a *P* < 0.05.

## Results

### Baseline characteristics and albumin distribution

**FIGURE 1:**
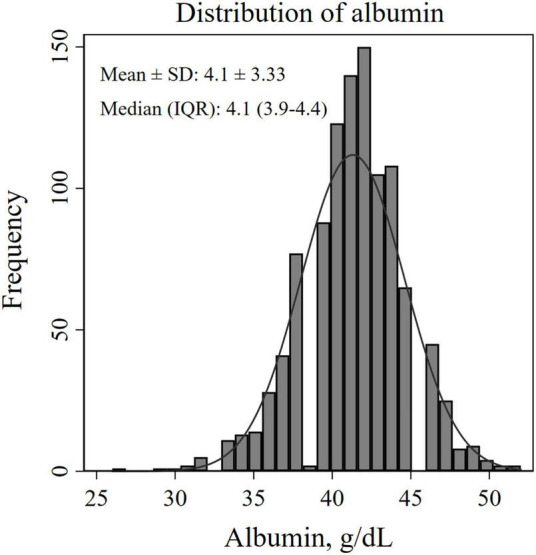
Histogram for estimating the distribution of albumin levels.

A total of 1,070 patients with CVD were included in the analysis, of which 156 deaths occurred during a median 34 months of follow-up. Patients in the lower tertiles were older, and more frequently observed in females. They also had a higher prevalence of diabetes and hypertension, and a higher mortality. Frequency distribution diagram showed an approximately normal distribution of albumin with an interval of 2.6–5.2 g/dl, with a median (IQR) of 4.1 (5) g/dl, and with a mean ± SD of 4.1 ± 3.33 g/dl. Details in Table 1 and Figure 1.

**TABLE 1:** Baseline characteristics of the study population stratified by albumin tertiles.

Factor	Tertile 1 (<4.1 g/dl)	Tertile 2 (4.1–4.3 g/dl)	Tertile 3 (>4.3 g/dl)	*P* for trend
*n*	404	398	268	
Age, year	67 ± 13	66 ± 13	64 ± 14	0.002
Female, sex, *n* (%)	234 (58)	168 (42)	95 (35)	<0.001
Ethnicity, *n* (%)				0.068
Mexican American	29 (7)	32 (8)	27 (10)	
Non-Hispanic White	192 (48)	218 (54)	147 (55)	
Non-Hispanic Black	128 (32)	87 (22)	40 (15)	
Non-Hispanic Asian	17 (4)	22 (6)	21 (8)	
Others	38 (9)	39 (10)	33 (12)	
BMI, kg/m^2^	32 ± 9	30 ± 7	28 ± 5	<0.001
Pulse, beats/min	71 ± 13	69 ± 12	71 ± 12	0.633
Drinking, *n* (%)				<0.001
Never	71 (19)	50 (14)	28 (11)	
Former	151 (40)	117 (32)	75 (31)	
Current	154 (41)	200 (54)	144 (58)	
Smoking, *n* (%)				<0.001
Never	177 (44)	156 (39)	114 (43)	
Former	133 (33)	152 (38)	99 (37)	
Current	94 (23)	90 (23)	55 (20)	
Hypertension, *n* (%)	131 (34)	111 (29)	67 (26)	0.016
Diabetes mellitus, *n* (%)	200 (50)	163 (41)	103 (38)	0.003
Triglyceride, mmol/L	1.6 ± 1.5	1.5 ± 1.1	1.6 ± 1.0	0.920
LDL-C, mmol/L	2.6 ± 0.9	2.6 ± 0.8	2.6 ± 1.0	0.793
HDL-C, mmol/L	1.3 ± 0.4	1.3 ± 0.4	1.4 ± 0.5	0.005
Albumin, g/L	38 ± 2	42 ± 0.8	45 ± 1.6	<0.001
Death cause, *n* (%)				
Cardiac deaths	28 (6.9)	15 (3.8)	5 (1.8)	<0.001
Hypertensive death	26 (6.4)	5 (1.3)	1 (0.3)	<0.001
Diabetic death	11 (2.7)	4 (1.0)	2 (0.7)	<0.001
Cancerous deaths,	7 (1.7)	9 (2.3)	5 (1.8)	0.008
Other caused deaths	9 (2.3)	17 (4.3)	12 (4.4)	<0.001
Deaths, *n* (%)	81 (20)	50 (13)	25 (9)	<0.001

Continuous and categorical variables were presented as mean ± SD or percentages *n* (%), respectively.

BMI, body mass index; LDL-C, low-density lipoprotein cholesterol; HDL-C, high-density lipoprotein cholesterol.

### The predictive role of albumin on long-term mortality in cardiovascular disease

**FIGURE 2:**
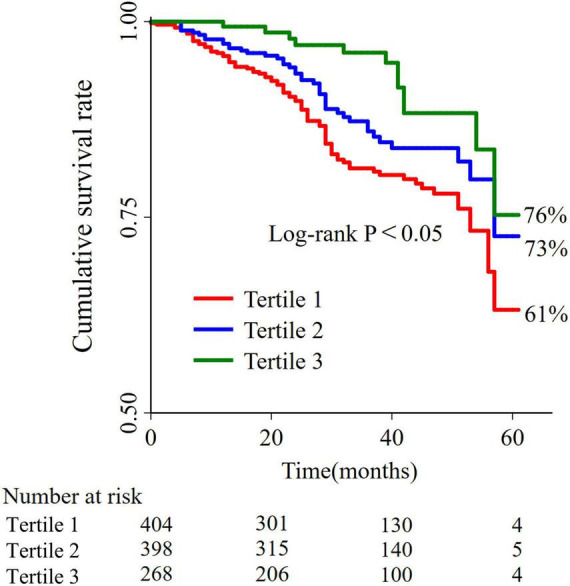
Kaplan-Meier survival curves for mortalities between different albumin tertiles.

The 5-year cumulative Kaplan–Meier survival were 61% (95% CI: 48–72%) for tertile 1, 73% (95% CI: 57–84%) for tertile 2, and 76% (95% CI: 51–89%) for tertile 3 (Figure 2). On a continuous scale, per 1 g/dl decrease was associated with an adjusted HR (95% CI) of 3.85 (2.38–6.25). On a categorical scale, as compared with tertile 3, the multivariable adjusted hazard ratio (95% CI) was 1.42 (0.74–2.71) for the tertile 2, and 2.24 (1.20–4.16) for the tertile 1, respectively, with respect to mortality. The *P*-values for trend test were <0.001. Details in Table 2.

**TABLE 2:** Association of albumin levels and cardiovascular disease (CVD) mortality in the COX model.

		Crude		Adjusted	
			
CVD mortality		HR (95% CI)	*p* for trend	HR (95% CI)	*p* for trend
On a continuous scale		4.55 (2.86–7.14)	<0.001*	3.85 (2.38–6.25)	<0.001*
Ona categorical scale	Tertile 1	2.98 (1.64–5.43)		2.24 (1.20–4.16)	
	Tertile 2	1.90 (1.00–3.59)	<0.001^†^	1.42 (0.74–2.71)	<0.001^†^
	Tertile 3	Reference		Reference	

Adjusted covariates: Age, sex, ethnicity, BMI, pulse, drinking, smoking, hypertension, diabetes mellitus, triglycerides, LDL-C, and HDL-C.

*per 1 g/dl decrease.

^†^per one tertile decrease.

### A J-shaped association of albumin levels with long-term mortality in cardiovascular disease

**FIGURE 3:**
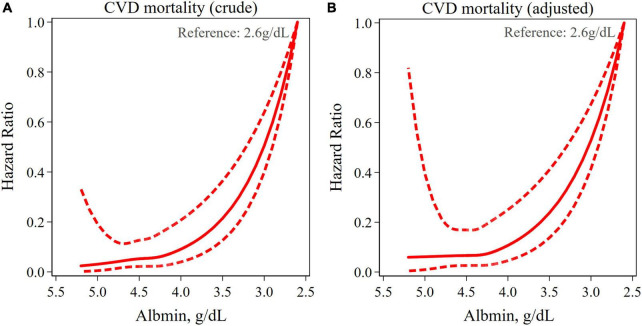
Restricted cubic spline (RCS) curves for analyzing the non-linear association between albumin levels and cardiovascular disease (CVD) mortality. Same variables adjusted as Table 2.

Non-linear analysis showed that the association between albumin levels and CVD mortality took on a J-shaped association, when the lowest albumin level was used as a reference (2.6 g/dl), as shown in Figure 3. The turning point for this J-shaped association was around 4.3 g/dl, beyond which, this association started to accelerate.

### Subgroup and sensitivity analysis

**FIGURE 4:**
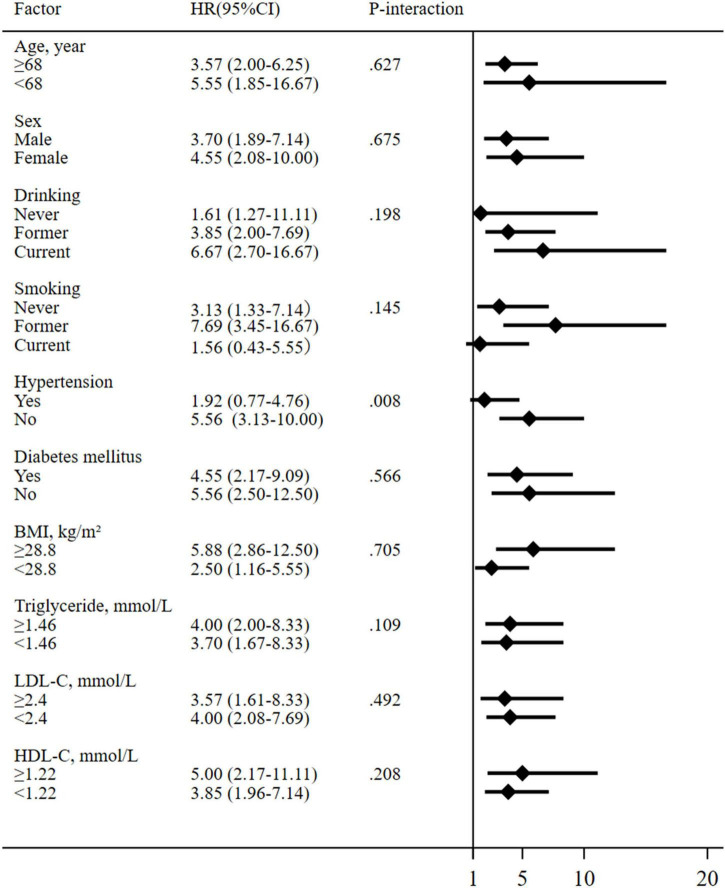
Subgroup analysis for the association between albumin levels and cardiovascular disease (CVD) mortality. Continuous variables were dichotomized at their corresponding medians. All models are adjusted as in Table 2.

Subgroup analysis largely confirmed the associations of albumin levels with CVD mortality revealed in the current study across a broad spectrum of risk factors as shown in Figure 4. To test the sensitivity of models, we selected participants from the 2011–2012 cycle for the same analysis and found a similar relationship between albumin levels and CVD mortality (data not shown).

## Discussion

The key findings revealed in the current study included that (i) to the best of our knowledge, the J-shaped albumin-CVD mortality association was first revealed; (ii) on a continuous scale or on a categorical scale, low albumin levels were significantly associated with higher CVD mortality, and this association remained substantially unchanged after adjusting for confounding factors; and (iii) the threshold effects were observed in terms of the albumin-CVD mortality association.

On a continuous scale, the current study showed an adjusted 3.85-fold increase in the risk of CVD death for every 1 g/dl decrease in albumin. A 3-year follow-up study demonstrated a 4.11-fold increase in the risk of death in patients with acute myocardial infarction for each 1 g/dl decrease in albumin in the univariate model, consistent with our findings (4). Another study described findings that for each 1/dl decrease in albumin, the risk of cerebro-cardiovascular death increased by 4.26-fold in the general population at 15 years of follow-up (19). Slightly higher than our findings. The reasonable explanations include 15-year long-term follow-up with more events recorded. On a categorical scale, previous studies also revealed an association between albumin tertiles and CVD mortality. Patients with acute coronary syndrome with albumin <3.5 g/dl had a 2.80-fold higher risk of death compared to those with albumin ≥3.5 g/dl [OR (95% CI), 2.80 (1.11 1–7.00)] (4). A 5-year follow-up of patients with systolic heart failure found a 2.2-fold higher risk of death from hypoalbuminemia (albumin ≤3.4 g/dl) compared to non-hypoalbuminemia [HR (95% CI), 2.2 (1.4–3.3)] (10). A large meta-analysis showed that during the 3- month follow- up period, compared with 4 to 4.49 g/dl group, patients with acute ischemic stroke in <3.5 g/dl group had a 2.13-fold higher risk of death [HR (95% CI), 2.13 (1.41–3.23)] (21). We reported adjusted hazard ratios (95% CI) of 1.42 (0.74–2.71) and 2.24 (1.20–4.16) for the tertile 2 (4.1∼4.3 g/dl) and for the tertile 1 (<4.1 g/dl), respectively, when compared to the tertile 3 (>4.3 g/dl), which are very similar to those aforementioned studies.

To the best of our knowledge, a J-shaped association between albumin and CVD mortality was first revealed in the current study. This J-shaped albumin-CVD association and the presence of a threshold effect may have potential implications for CVD prevention and treatment: in the steep limb of the J-shaped curve, a slight change in the serum albumin levels will dramatically increase or decrease the risk of CVD death; by contrast, in the almost horizontal limb of the J-shaped curve, infusion of albumin to achieve a higher level of serum albumin (beyond the threshold) will not further result in the reduction of CVD death. The J-shaped predictive effect of serum albumin levels in CVD mortality may be mainly related to its irreplaceable physiological functions, including its effects on antitumor, anti-inflammatory, antioxidant and antithrombotic activity. Decreased albumin levels weaken the antioxidant capacity of the vascular endothelium, initiate and exacerbate the inflammatory process, increase blood viscosity, and increase the risk of thrombosis, all of which, in combination or separately, contribute to the poor prognosis of CVD (25–28).

## Limitations

Several limitations of the current study should be discussed. First, the design of the study was limited in that not all patients obtained serum albumin values at the same time interval and the etiology of low albumin levels was not distinguished; Second, some of the covariates selected for this study such as smoking, drinking, hypertension, and diabetes were obtained in combination with subjective questionnaires, which may bias the results; Third, our study was conducted on patients in the general U.S. population, so it is not clear whether the J-shaped predictive effect of albumin is also applicable to other populations or ethnic groups.

## Conclusion

A J-shaped association between low serum albumin levels and increased long-term mortality of CVD has been revealed. This J-shaped association’s implications for CVD prevention and treatment are deserving of being further studied.

## Data availability statement

The datasets presented in this study can be found in online repositories. The names of the repository/repositories and accession number(s) can be found in the article.

## Ethics statement

This survey protocol was approved by the Research Ethics Review Board of the NCHS and all participants have signed and provided informed consent.

## Author contributions

XL and YZ wrote the main manuscript. W-XC and Y-MH provided statistical guidance and were responsible for the final revisions. YH, K-XL, R-NX, HW, and T-BJ provided graphic art direction and supported the successful completion of the work. All authors contributed to the article and approved the submitted version.

## Abbreviations

NHANES: National Health and Nutrition Examination Survey
CVD: cardiovascular disease
CHD: coronary heart disease
HF: heart failure
BM: body mass index
LDL-C: low-density lipoprotein cholesterol
HDL-C: high-density lipoprotein cholesterol
SBP: systolic blood pressure
DBP: diastolic blood pressure
ADA: American Diabetes Association
A1C: glycated hemoglobin
FPG: fasting plasma glucose
OGTT: oral glucose tolerance test
SD: Standard deviation
HR: Hazard ratio
CI: confidence interval.
